# Temperature-Controlled 3D Cryoprinting Inks Made of Mixtures of Alginate and Agar

**DOI:** 10.3390/gels9090689

**Published:** 2023-08-26

**Authors:** Leo Lou, Boris Rubinsky

**Affiliations:** 1 Department of Bioengineering, University of California Berkeley, Berkeley, CA 94720, USA; taolou@berkeley.edu; 2 Department of Mechanical Engineering, University of California Berkeley, Berkeley, CA 94720, USA

**Keywords:** temperature-controlled cryoprinting, alginate–agar gel, microstructure, dual solidification

## Abstract

Temperature-controlled 3D cryoprinting (TCC) is an emerging tissue engineering technology aimed at overcoming limitations of conventional 3D printing for large organs: (a) size constraints due to low print rigidity and (b) the preservation of living cells during printing and subsequent tissue storage. TCC addresses these challenges by freezing each printed voxel with controlled cooling rates during deposition. This generates a rigid structure upon printing and ensures cell cryopreservation as an integral part of the process. Previous studies used alginate-based ink, which has limitations: (a) low diffusivity of the CaCl_2_ crosslinker during TCC’s crosslinking process and (b) typical loss of print fidelity with alginate ink. This study explores the use of an ink made of agar and alginate to overcome TCC protocol limitations. When an agar/alginate voxel is deposited, agar first gels at above-freezing temperatures, capturing the desired structure without compromising fidelity, while alginate remains uncrosslinked. During subsequent freezing, both frozen agar and alginate maintain the structure. However, agar gel loses its gel form and water-retaining ability. In TCC, alginate crosslinking occurs by immersing the frozen structure in a warm crosslinking bath. This enables CaCl_2_ diffusion into the crosslinked alginate congruent with the melting process. Melted agar domains, with reduced water-binding ability, enhance crosslinker diffusivity, reducing TCC procedure duration. Additionally, agar overcomes the typical fidelity loss associated with alginate ink printing.

## 1. Introduction

Organ transplantation remains the sole treatment option for individuals with end-stage organ failure [1]. However, the scarcity of available donor organs, coupled with logistical challenges arising from the limited ex vivo organ survival time, results in a significant number of patients dying before they can receive a transplant [2,3]. The pressing issue of organ shortage has propelled research in the field of 3D bioprinting, offering a promising solution to alleviate the crisis [4].

Three-dimensional bioprinting is an additive manufacturing technology that uses a digital file as a blueprint to print an object, voxel by voxel and layer by layer. Although tissue engineering was introduced by Langer and Vacanti approximately 30 years ago [5], the technology for 3D printing large organs is still in its developmental stages and has not reached maturity [6]. A notable accomplishment in 3D bioprinting is the successful fabrication of a functioning miniature heart at a scale of 15 mm [7]. The fabrication of the miniature heart was achieved through a four-hour long process, which underscores both the progress made in bioprinting complex organs and the challenges that remain in scaling up the technology for larger organs. The storage of large organs post-3D bioprinting parallels the challenges currently faced in preserving donor organs for transplantation. Curiously, this specific challenge has not received any attention from researchers focused on large organ production via 3D bioprinting.

To develop effective 3D bioprinting technologies for the fabrication of large organs, it is important to understand the limitations in current 3D bioprinting methods. A commonly used 3D bioprinting technology is extrusion printing [8], in which a bioink is extruded through a nozzle and deposited voxel by voxel, layer by layer, to construct the desired 3D structure [9]. One of the pivotal elements in 3D bioprinting is the composition of the bioink, which comprises a combination of cells, biomaterials, and bioactive molecules. Aqueous solutions of sodium alginate are commonly used as bioprinting inks [8]. This is due to alginate’s exceptional biocompatibility, biosafety, and cost-effectiveness [10]. Alginate is extracted from the cell walls of brown algae in the form of alginic acid. Sodium alginate, derived from alginic acid, can form hydrogels by substituting the sodium ions from the guluronic acid residues with different divalent cations, such as ${Ca}^{2+}$, which crosslink the polymer chains via the “egg-box” model [11]. The crosslinking of alginate is irreversible.

In 3D bioprinting, the viscosity of the ink plays a crucial role. The low viscosity of alginate before crosslinking enables easy extrusion through a small nozzle, facilitating high precision and resolution during printing. Moreover, the use of low-viscosity ink is vital for cell viability within the extruded ink. Studies have indicated that cells do not survive extrusion with high-viscosity inks [12]. However, maintaining the structural rigidity of alginate-based 3D printed objects requires crosslinking. This presents a challenge in determining the optimal timing for crosslinking: if performed before printing, the alginate becomes too viscous to be extruded through the nozzle. Delaying crosslinking for too long after deposition on the print plate can result in the collapse of the non-crosslinked alginate structure. Also, even in its crosslinked form, the 3D printed alginate structure is not sufficiently rigid to support the weight of a large organ during the layer-by-layer deposition process, prior to the completion of the structure. This limitation poses a significant challenge in the 3D bioprinting of large-scale structures. In the bioprinting of large organs, living cells are included within the bioprinting ink. However, a significant challenge arises in ensuring the survival of these 3D bioprinted cells throughout the lengthy printing process. As previously mentioned, the 3D bioprinting of a miniature heart typically takes around four hours. Unfortunately, cells cannot remain viable outside their optimal growth environment for such prolonged durations. Consequently, it is likely that the cells within the initially printed layers may not survive until the completion of the printing process. This cell survival challenge presents a critical hurdle in the successful bioprinting of large organs.

**Figure 1:**
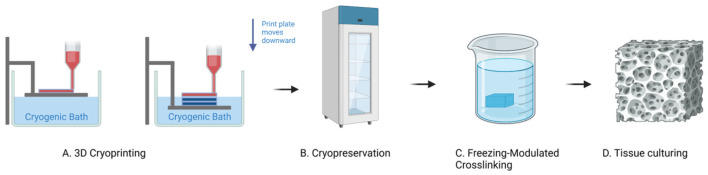
The process of temperature-controlled cryoprinting involves several key steps: (**A**) After each layer is printed, the printing surface is immersed in a cooling bath. This immersion helps maintain a controlled distance between the top surface of the cryogen bath and the last printed layer. By carefully controlling this distance, the freezing process can be controlled and optimized for the desired outcomes. (**B**) Once the printing process is complete, the frozen product is stored in a −80 °C freezer. This low-temperature storage helps preserve the integrity and stability of the printed structure until further use or analysis. (**C**) Freezing-modulated crosslinking is achieved by immersing the printed product in a CaCl_2_ bath at room temperature. This step allows for the crosslinking of the printed materials, further enhancing the structural stability and integrity of the construct. (**D**) Studies have indicated that cells can survive the temperature-controlled cryoprinting process. The careful control of freezing and thawing conditions, along with the preservation of cell viability during the printing and crosslinking steps, contributes to the successful survival of cells within the printed constructs. Ongoing research continues to explore and optimize this process to ensure the best possible outcomes for cell viability and functionality [12].

Our research group has made significant advancements in addressing the challenges associated with 3D bioprinting of large structures through the development of the “Temperature-Controlled-Cryoprinting” (TCC) technology [13,14,15]. We utilized low-viscosity alginate solutions as the 3D printing ink, which facilitated precise ink deposition and improved cell survival [12]. The key innovation in the TCC technology resides in the precise temperature control during the freezing of each deposited voxel as it is deposited [13,15]. Figure 1 provides an illustration of the TCC process [16]. It shows that as each layer is printed and frozen, the platform is submerged in a cryogenic fluid bath maintained at a controlled temperature below freezing. In the TCC process, the distance between the last printed and frozen layer and the cryogenic bath upper surface is carefully regulated to enable freezing with a controlled cooling rate for the subsequent printed layer.

Freezing of each layer as it is deposited imparts immediate rigidity to the structure as soon as it is printed. Consequently, it enables the manufacturing of complex and large structures that would otherwise be challenging to achieve. For instance, the creation of structures with internal cavities becomes feasible through this method [17]. Freezing of a gel with temperature-controlled cryoprinting, has several other advantages in addition to providing rigidity to the printed object. Conventional 3D printing generates isotropic structures at the microscale. Temperature-controlled freezing can be used to control the size, structure, and direction of the ice crystals that form [6,7,8]. This can be used to generate controlled anisotropic microstructures, anisotropic rigidity, and material properties of the 3D cryoprinted structure [3,11,12].

The accurate control of the cooling rate during the freezing of each layer provides another advantage. It allows the freezing of every deposited cell with optimal cryopreservation thermal parameters during the entire 3D bioprinting process [14,18]. With TCC, each cell in the entire structure is cryopreserved as soon as it is printed [12]. Once the entire 3D structure is frozen using temperature-controlled cryoprinting (TCC), all the cells within the large structure are cryopreserved using optimal cryopreservation protocols. This ensures their viability and makes the structure ready for storage in a frozen state and transportation. This is particularly important in printing large biological organs with cells, because 3D printing is a slow process and cells printed in the early stage of the process can die by the time the entire organ is printed. Recent studies have demonstrated the capability of TCC technology to produce large, cryopreserved 3D printed structures with cells that successfully survive in the printing process and subsequent cryopreservation [12]. In that study, the bioprinting ink used also incorporated a cryoprotective solution, specifically, dimethyl sulfoxide (Me_2_SO). The inclusion of Me_2_SO in the bioprinting ink played a crucial role in ensuring the long-term survival of cells within the 3D printed structure during cryopreservation at a temperature of −80 °C. This finding highlights the effectiveness of the TCC-enabled cryopreservation process and the potential for extended storage of 3D bioprinted structures with preserved cell viability.

In the temperature-controlled-cryoprinting (TCC) technique, the crosslinking of alginate with CaCl_2_ can be performed through two methods: before freezing or after freezing, during thawing [19]. This study specifically focuses on the technique where crosslinking occurs during the thawing process [16]. In this approach, the entire object is 3D printed using temperature-controlled cryoprinting of each alginate voxel before any crosslinking takes place. The frozen structure (with or without cells) is maintained at a desired subfreezing preservation temperature [12,16]. During the crosslinking step, the frozen structure is immersed in a solution of a crosslinker, typically CaCl_2_, at a controlled temperature, above freezing. The temperatures of the frozen object and the crosslinking bath are carefully chosen to ensure that as the frozen object melts, the diffusion of CaCl_2_ into the melted region occurs simultaneously with the propagation of the change of phase interface [16]. This allows for crosslinking to take place as the structure melts, thereby achieving structural rigidity [16]. A schematic representation of all steps involved in this procedure is illustrated in Figure 1 ([12]).

The crosslinking technique described above has its limitations. The propagation of the change of phase interface and the diffusion of the CaCl_2_ must occur simultaneously to ensure that the structure’s rigidity, obtained through freezing, is immediately replaced by the rigidity provided by alginate crosslinking. The melting process is affected by the lower diffusivity of CaCl_2_ through the crosslinked alginate compared to thermal diffusivity. It would be beneficial to discover methods to enhance the diffusivity of CaCl_2_ through the crosslinked alginate, thereby reducing the time required for this manufacturing step. Additionally, this technology shares a common drawback with all alginate-based 3D printing processes, which is the shrinkage of the printed structure. This shrinkage leads to a decrease in form fidelity [16]. This study was specifically conducted to address the two beforementioned limitations: (a) the low diffusivity of CaCl_2_ through crosslinked alginate during the melting-associated crosslinking process and (b) the commonly observed low form fidelity in alginate-based 3D printing.

This study aimed to investigate the hypothesis that incorporation agar into the alginate ink could potentially enhance the diffusion of the CaCl_2_ during the melting/crosslinking step, resulting in better maintenance of shape fidelity. A recent observation on the freezing and melting of agar gel is particularly relevant [20]. According to the study, the ice crystals formed during the freezing of an agarose gel disrupt the structural integrity of the gel. Upon melting, the gel structure transforms into alginate flakes and free water.

A rough comparison between the diffusion coefficient of CaCl_2_ in an aqueous solution [21] and in a 2% agar gel [22] reveals that the diffusion in the aqueous solution is higher than in the gel by a factor of about 1.5. An order-of-magnitude comparison between the diffusion coefficient of CaCl_2_ in aqueous solutions and the diffusivity of ${Ca}^{2+}$ crosslinked alginate [23] demonstrates that the diffusivity in an aqueous solution is at least one order of magnitude greater than that in the crosslinked alginate. It is important to note that these values are not precise and depend on various factors, particularly the concentrations of agar and alginate. Nevertheless, these findings suggest that the addition of agar to an alginate ink should increase the diffusivity of CaCl_2_ through the crosslinked alginate after the melting of the frozen agar gel.

The practice of adding agar to alginate to enhance the rigidity and printing fidelity of alginate in casting and 3D printing is well documented. In fact, the use of a combination of agar and alginate for casting dental imprints has been known since 1937. Extensive research in the field of dentistry has demonstrated that agar produces imprints that closely resemble the source, while the combination of agar and alginate results in a gel that is easier to handle, possesses mechanical strength like alginate, and reliably captures impressions, as evidenced in [24]. Agar has been employed in conjunction with alginate in various other applications. For instance, agar was incorporated into alginate hydrogel beads for oral drug delivery, aiming to enhance the mechanical strength of the beads [25]. The combination of agar and alginate has also been utilized in the production of packaging films [26]. Furthermore, this combination has already found utility in 3D bioprinting, where it increases ink viscosity and imparts rigidity to the printed objects [27,28,29]. Based on these previous applications, we envisioned that an ink composed of agar and alginate could retain the fidelity of the print, even when using the TCC (melting/crosslinking) method.

Agar is a gel-like substance derived from the cell walls of red algae. Unlike alginate, which forms gels through chemical crosslinking, agar gels undergo physical changes, specifically hydrogen bonding crosslinking. Agar transforms into a gel as it cools from 85 °C to about 32–42 °C. Notably, the gelation of agar is reversible, unlike the irreversible crosslinking experienced by alginate.

In the envisioned TCC process using an ink of agar and alginate, the following sequence of events takes place when a solution of agar and alginate is deposited on the printing surface. As the printed voxel cools to approximately 30 °C, the agar first undergoes gelation by forming a network through hydrogen bonding crosslinking of agar molecular chains. At this stage, the alginate is not yet crosslinked but is trapped within the agar gel network, with the structure of the print being fixed by the agar gel. During TCC, the deposited voxel freezes completely, encompassing the gelled agar and the liquid alginate held in place by the agar network. As the frozen object begins to thaw in a solution of CaCl_2_, as described in [16], the molten agar loses its structural integrity and assumes the diffusivity characteristics of water instead of that of a gel, as previously discussed. Consequently, the diffusivity of CaCl_2_ through the crosslinked alginate with liquid agar becomes higher compared to the diffusivity in a crosslinked alginate solution without agar. This results in faster and more controlled crosslinking. The fidelity of the printed structure is initially maintained during TCC through the gelation of agar, followed by freezing, and ultimately through the crosslinking of the alginate.

In this paper, we will explore the effects of the agar/alginate ink composition on the TCC process regarding deformation, diffusivity, stability in an environment that mimics the human body, microscale structure, and printability.

## 2. Results and Discussion

### 2.1. Structure Retaining Test

#### 2.1.1. Crosslinking Test

**Figure 2:**
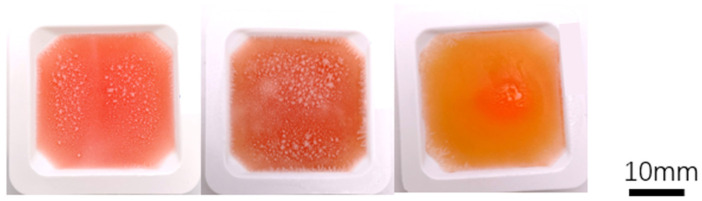
The casted samples in the tray are arranged from left to right as follows: 2% SA/2% AG, 2% SA/1% AG, and 2% SA. These compositions represent the different agar/alginate combinations used in the study. The tray provides a clear visual representation of the samples, allowing for a side-by-side comparison of their appearance and potential differences in shape, size, or other characteristics. By arranging the samples in this manner, readers can easily observe and analyze the variations between the different compositions and their resulting physical properties.

Hydrogel solutions containing 2% SA, 2% SA/1% AG, and 2% SA/2% AG were cast in the tray shown in Figure 2. To enhance visibility and highlight the shape of the hydrogels, 1 mL of red food dye from Betty Crocker (Minneapolis, MN, USA) was added per 100 mL of gel solution. The cast hydrogel samples were then placed in a −80 °C freezer for a duration of 30 min to initiate freezing and solidification. Following the freezing step, the samples were transferred to a 2% CaCl_2_ bath at room temperature (23 °C). This bath facilitated the melting of the hydrogels while simultaneously promoting crosslinking. By subjecting the frozen hydrogel samples to the room-temperature CaCl_2_ bath, the freezing was reversed, allowing the hydrogels to melt and crosslink simultaneously, resulting in the formation of structurally stable hydrogel constructs.

**Figure 3:**
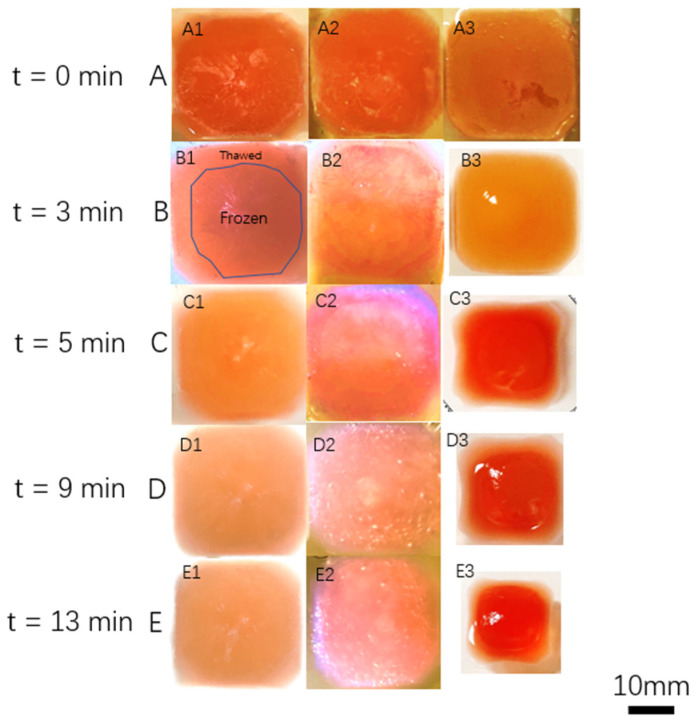
The thawing and crosslinking process for the cast samples is visually depicted. From left to right, the cast samples include 2% SA/2% AG, 2% SA/1% AG, and 2% SA. As shown in **B1**, an example image is provided to differentiate the thawed (gel-like) and frozen (ice-crystal) areas within the cast samples. In **C1**, the ice crystal within the 2% SA/2% AG sample completely disappears when the elapsed time reaches 5 min. This indicates the successful thawing and transformation of the frozen region into a gel-like structure. Moving to **D2**, the ice crystal within the 2% SA/1% AG sample disappears at 9 min of elapsed time. This signifies the completion of the thawing process for this specific sample. Similarly, in **E3**, the ice crystal within the 2% SA sample also disappears at 9 min of elapsed time. This indicates the successful thawing and transformation of the frozen region into a gel-like structure. These visual observations demonstrate the progressive thawing and disappearance of ice crystals within the cast samples, signifying the completion of the crosslinking and thawing process.

In Figure 3, the top view of the cast samples is presented after they were removed from the freezing tray and immersed in the crosslinking bath. The figure illustrates the progression of time for each of the three combinations of alginate and agar inks. In Figure 3A, the sample’s appearance is depicted immediately upon immersion in the crosslinking bath (t = 0 min). The dimensions of the sample are labeled as x–y. Moving on to Figure 3B, the cast sample is displayed after 3 min of immersion in the crosslinking bath. The outer dimensions of both the 2% SA/1% AG and 2% SA/2% AG samples measure 3.5 cm × 3.5 cm. However, the 2% SA sample exhibits shrinkage, resulting in outer dimensions of 3 cm × 3 cm. Additionally, the region that has melted has become translucent, indicating the transition from the frozen solidified state to a gel-like form. Continuing with Figure 3C, the appearance of the cast samples is depicted 5 min after immersion in the crosslinking bath. The outer dimensions of the AG groups (2% SA/1% AG and 2% SA/2% AG) remain constant at 3.5 cm × 3.5 cm. However, the 2% SA sample continues to experience shrinkage, resulting in further reduction in its outer dimensions. At this point, the 2% SA sample reaches outer dimensions of 2.7 cm × 2.7 cm. Remarkably, in the 2% SA/2% AG cast, the frozen region has completely disappeared by this stage. This suggests that the crosslinking process is effectively transforming the frozen gel into a structurally stable hydrogel, resulting in the disappearance of the initially frozen region. Indeed, the observations presented in Figure 3 support the hypothesis that the addition of agar to the 3D printing ink enhances the diffusion of the crosslinker into the melting gel. The faster melting process observed in the agar-containing samples suggests that agar facilitates the penetration of the crosslinker throughout the gel matrix. It is also plausible that the faster melting can be attributed to the higher thermal diffusivity of water compared to the crosslinked alginate matrix. Water is known to have a higher thermal diffusivity, meaning it conducts heat more efficiently. As a result, the heat from the crosslinking bath is more readily transmitted through the water-rich agar gel, leading to faster melting and crosslinking reactions. These findings highlight the positive impact of agar on diffusion and melting properties, potentially improving the overall performance and stability of the hydrogel structures fabricated using the TCC process. In Figure 3D, the samples are shown 9 min after the start of crosslinking. The inner and outer dimensions of the AG-containing samples (2% SA/1% AG and 2% SA/2% AG) remain unchanged at 3.5 cm × 3.5 cm, indicating their structural stability. However, the 2% SA ink continues to experience shrinkage, resulting in further reduction in dimensions. At this stage, the 2% SA sample reaches dimensions of 2.4 cm × 2.4 cm, indicating a significant decrease in size compared to the initial cast. Additionally, in the 2% SA/1% AG cast, the frozen core completely melts by this time. This suggests that the crosslinking process and the diffusion of the crosslinker throughout the gel have effectively converted the frozen core into a gel-like structure, leading to the disappearance of the initially frozen region.

Indeed, when comparing Figure 3C,D, it becomes apparent that the concentration of agar in the hydrogel ink influences the crosslinking process. A higher concentration of agar leads to a larger amount of free water within the gel matrix upon melting. As a result, the increased presence of free water facilitates the diffusion of the crosslinker throughout the gel, accelerating the crosslinking process. Consequently, the higher concentration of agar reduces the overall crosslinking time, as observed in Figure 3D compared to Figure 3C. This finding suggests that the concentration of agar in the hydrogel ink can be manipulated to control the crosslinking kinetics and tailor the properties of the resulting hydrogel structures.

In the final stage (Figure 3E), at 13 min after the start of crosslinking, all the samples have completed the thawing process. The outer dimensions of the groups containing agar (2% SA/1% AG and 2% SA/2% AG) are retained, indicating their structural integrity and shape retention. However, the groups composed of 2% SA lose their original shape, undergoing a transformation into irregular square shapes with dimensions of 2 cm × 2 cm. This suggests that the crosslinking and melting processes have caused significant deformation and restructuring of the 2% SA samples, resulting in the loss of their initial shape. Indeed, the observations highlight the significant value of incorporating agar into the alginate ink to enhance the structural stability and shape retention of hydrogel constructs during the crosslinking and thawing processes. The addition of agar to the alginate ink increases the viscosity of the printing ink, which can contribute to improved shape fidelity and structural integrity [28].

However, it is essential to consider the impact of ink viscosity on the survival and viability of living cells within the hydrogel constructs. Future studies should focus on optimizing the viscosity of the ink to strike a balance between structural stability and cell viability. Finding the appropriate viscosity will be crucial to ensure optimal printing conditions that support the viability and functionality of living cells within the printed constructs. By fine-tuning the ink viscosity, it may be possible to achieve both excellent structural stability and the necessary conditions for cell survival, leading to improved outcomes in tissue engineering and other related applications.

**Figure 4:**
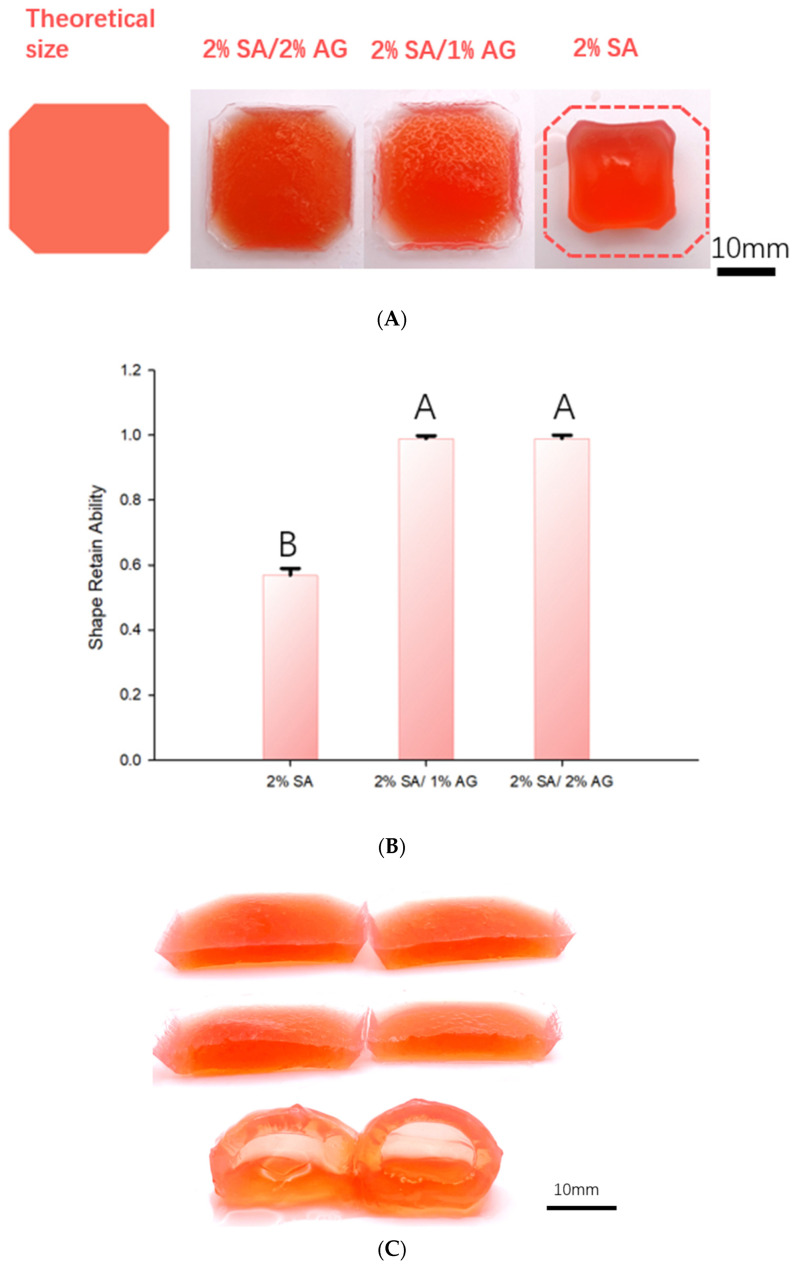
(**A**) Top view of samples after 15 min of crosslinking: Figure **A** displays a top-down view of the samples, showcasing the shape and appearance of each scaffold after the crosslinking process. The 2% SA scaffold (foremost right—the dashed outline is the original intended shape) exhibits noticeable deformation and a loss of its original structure. In contrast, the SA/AG groups (the two from the left) maintain their intended shapes with minimal deformation. (**B**) Shape retention ability: Figure **B** represents a visual representation in the form of a graph, illustrating the shape retention ability of the scaffolds. It provides a comparison of the different compositions, as bar graphs, showing the calculated shape retaining ability values for each scaffold. The graph allows for an understanding of the relative shape retention abilities among the different groups. (**C**) Cross section of sample 9 min after immersion in the CaCl_2_ bath. Top: 2% Sa/2% AG; middle: 2% SA/1% AG; bottom: 2% SA.

Following a crosslinking duration of 15 min, the scaffolds underwent subsequent measurements to assess shape alterations. Figure 4A illustrates the shapes of the scaffolds after crosslinking, juxtaposed with the initially intended outlines (indicated by dashed lines). Clearly, the 2% SA scaffold encountered significant distortion, veering away from its originally planned configuration. In contrast, the SA/AG groups exhibited commendable shape preservation, effectively upholding their intended structures. These tests were carried out in triplicate.

Figure 4B illustrates the shape retention capabilities of the scaffolds through graphical representation. The quantitative assessment of shape retention was defined by the following expression:

Shape Retaining Ability = (Final Dimension/Initial Dimension)

This measure gauges the scaffolds’ capacity to uphold their intended shapes. The evaluation was conducted by calculating the ratio of the final cross-sectional area of the scaffold following crosslinking to its initial cross-sectional area before undergoing crosslinking. This computation yields a numerical value signifying the degree to which the scaffold maintained its form after the crosslinking procedure.

The visual depiction in Figure 4B enables a comparative analysis of the shape retention abilities among the various compositions, providing insight into the effects of different formulations. The testing was executed in triplicate.

Figure 4C offers further insights into both the crosslinking process and the diffusivity of CaCl_2_ within the crosslinked alginate. This is achieved by presenting cross-sectional views of samples created with varying ink compositions. These views were captured 9 min after the samples were immersed in the crosslinking bath. In both the 2% SA and 2% SA/1% AG samples, an inner core of gelatinous liquid is noticeable within the structure. This gelatinous core develops due to the pre-crosslinking melting of the central region of the frozen object. However, upon the introduction of agar, the size of this viscous, liquid-like core is diminished. In the case of the 2% agar sample, no liquid core is evident whatsoever. This phenomenon can be attributed to the impact of agar on crosslinker diffusivity following its melting and the subsequent release of bound water. The presence of agar alters the diffusion rate of the crosslinker within the gel matrix. The existence or absence of a liquid core among the different ink compositions yields invaluable insights into the crosslinking mechanism and the distribution of the crosslinker within the gel matrix. This disparity underscores the role of agar in modifying crosslinker diffusivity and its influence on the overall structure and characteristics of the resultant hydrogel constructs. The temperature-controlled cryoprinting (TCC) technology hinges on the concurrent diffusion of the crosslinker and the melting process to uphold the frozen structure. Should melting take place prior to the crosslinker’s arrival at the melting interface, it results in a configuration featuring an exterior flexible rim encompassing an inner liquid core. In the case of the 2% SA sample, the phenomenon of surface tension prompts the object to minimize its outer surface area, consequently causing it to swell into a spherical shape. This effect aligns with findings from other research which has also delved into the swelling phenomenon within the crosslinking process of sodium alginate scaffolds [30]. The non-linear swelling behavior stands as an undesirable occurrence within the realm of bioprinting with alginate. This study demonstrates that the inclusion of agar in the ink can effectively mitigate this unfavorable outcome.

**Figure 5:**
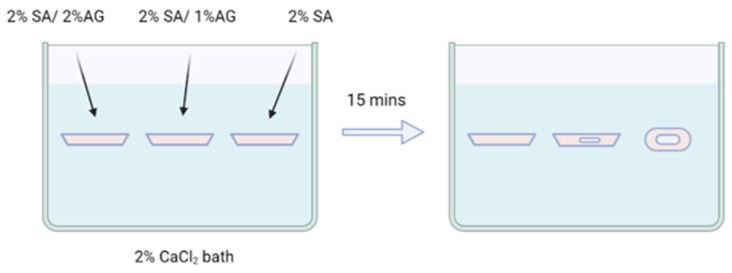
Schematic of the crosslinking process for hydrogels with different compositions. The figure shows in a schematic the changes in the cross section of a cast after 15 min exposure of the frozen sample to the crosslinking bath. It is important to observe that the 2% SA/1% AG and the 2SA have a liquid core (the internal enclosure in the schematic). However, the 2% SA/1% AG has retained the original shape, while the 2% SA has taken a rounded exterior shape.

Figure 5 provides a schematic illustration of the experimental results in Figure 4C. The solid scaffolds fabricated using different concentrations of agar/alginate exhibit varying levels of deformation during the thawing/crosslinking process. The deformation is affected by the diffusivity of the crosslinker. In the thawing sample of 2% SA, the diffusivity of the crosslinker is low due to the tortuosity caused by the crosslinked alginate and the trapped water molecules. However, the addition of agar, which loses its ability to bind water during thawing, reduces the tortuosity during melting and increases the diffusivity of the crosslinker through the molten part. It is well known that the diffusivity within a crosslinked gel is significantly lower compared to that in water [31]. Therefore, the higher concentration of agar, which upon melting yields more free water, leads to higher diffusivity. These observations highlight the crucial role of agar in controlling the diffusion of the crosslinker and preserving the desired structure during the crosslinking process in TCC.

Overall, the addition of agar influences the diffusivity, reduces tortuosity, and plays a critical role in controlling the diffusion of the crosslinker, ultimately preserving the desired structure during the crosslinking process in TCC.

#### 2.1.2. Liquid Exposure Test

To address the challenge of retaining shape in vivo faced by traditional scaffolds made from naturally derived polymers like collagen/gelatin, alginate, fibrin, and hyaluronic acid, an experiment was conducted to assess the structural integrity of agar/alginate scaffolds fabricated using the temperature-controlled cryoprinting (TCC) technique under liquid exposure conditions. The experimental setup and scaffold geometries were consistent with the previous post-crosslinking shape test. This experiment aimed to evaluate how the agar/alginate scaffolds perform in terms of maintaining their structural integrity when exposed to a liquid environment. The liquid exposure condition simulates the in vivo environment, allowing researchers to assess the scaffold’s ability to retain its shape and structural stability. By conducting this experiment, valuable insights can be gained regarding the suitability of agar/alginate scaffolds for tissue engineering applications, specifically in terms of their shape retention and structural integrity under liquid exposure.

**Figure 6:**
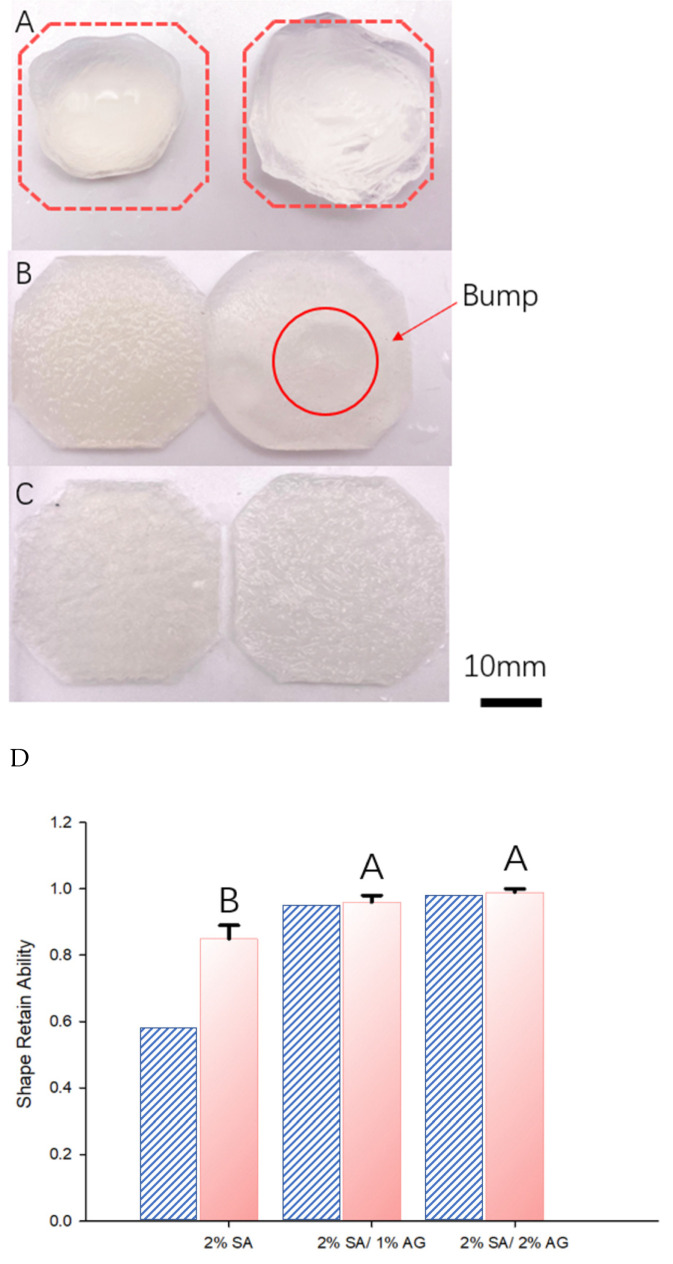
The 24 h 1× PBS exposure test (no food dye added). Left samples: samples kept in the Ziploc bag in 4C refrigerator. Right samples: samples immersed in the 1× PBS. (**A**) 2% SA (dashed outline: original intended shape). The scaffold expanded and became deformed after 24 h of exposure. (**B**) 2% SA/1% AG. A bump was noticed after 24 h of exposure in the 1× PBS. This is attributed to the tiny liquid core at the center (**C**) 2% SA/2% AG. With the concentration of agar increase, the liquid core in the center disappeared (**D**) Shape retaining ability.

Figure 6 displays the results of the liquid exposure test conducted on the ink-fabricated scaffolds. For each ink composition, two samples were prepared: one stored in a sealed Ziploc bag at 4 °C, and the other immersed in a 1×PBS solution to simulate the body environment. All samples were kept for 24 h. Figure 6A–C show the appearance of the samples after liquid exposure. The dashed-line schematic in Figure 6A shows the original intended shape and is superimposed on the photographs of the sample to facilitate visual comparison. Figure 6D illustrates the shape retain ability of the different samples, according to the shape retention formula, after casting (dashed lines) and after liquid exposure (orange).

The results indicate that only the 2% SA group exhibited a noticeable change in shape during the liquid exposure. Figure 6D shows that the dimensions of the samples from that group increased from 2 cm × 2 cm to 3 cm × 3 cm. In contrast, Figure 6D shows that both the 1% Agar/2% SA and 2% Agar/2% SA groups maintained their shape with no significant differences. However, it was observed that the 2% SA/1% AG sample exhibited a cavity-like liquid structure. This phenomenon is illustrated in the schematic diagram in Figure 5 and depicted in Figure 6. It is possible that the core of the cast object retained its liquid state and started absorbing liquid during storage. Prolonged exposure to the liquid environment caused the 2% SA group to become soft to the touch and altered its surface properties.

The shape retaining ability of the scaffolds was assessed in three replicates, ensuring the consistency and reliability of the results. These findings demonstrate the differential performance of the scaffold compositions under liquid exposure, with the 2% SA group showing significant shape changes, while the 1% Agar/2% SA and 2% Agar/2% SA groups maintained their structural integrity.

### 2.2. Electron Microscopy

The gelation mechanism of agar solutions has been widely studied and discussed in previous publications, such as reference [32]. These studies provide insights into the process by which agar chains in an aqueous solution undergo a coil/helix transition, ultimately forming a gel network through the extensive aggregation of helices during the cooling process. The gelation process involves both intra- and inter-molecular associations, with various molecular groups in the agar structure playing key roles. For example, the hemiacetal oxygen, hydroxyl, or methyl groups of the sugar residues in agar contribute to hydrogen bonding interactions, which are essential for gel formation [33].

**Figure 7:**
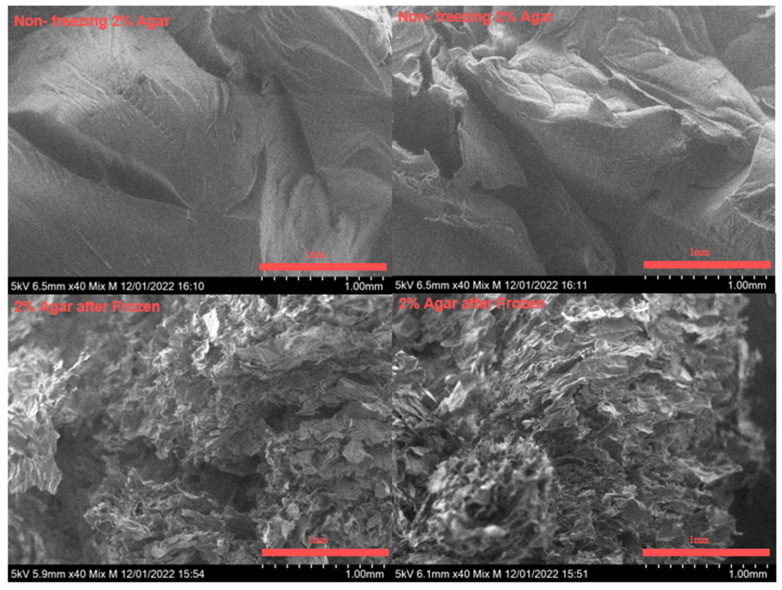
SEM images for 2% agar before (**top**) and after (**bottom**) freezing.

In Figure 7, electron microscope (ECM) images are presented to illustrate the difference between a structure of 2% agar gel before and after freezing. The images clearly show that the freezing process disrupts the structural integrity of the agar gel, and this integrity is not recovered upon thawing. The agar gel undergoes significant structural changes and loses its original organization during the freezing and thawing process. However, in the studied composite agar/alginate gel, the structural integrity is maintained upon thawing. This preservation of structural integrity is attributed to the presence of the crosslinked alginate component. The crosslinked alginate acts as a stabilizing agent, compensating for the loss of integrity observed in agar upon freezing and thawing. These findings highlight the role of alginate in preserving the structural integrity of the composite gel. By incorporating alginate into the gel matrix, the composite gel exhibits enhanced stability and resilience, enabling it to withstand the freezing and thawing processes while maintaining its structural integrity.

**Figure 8:**
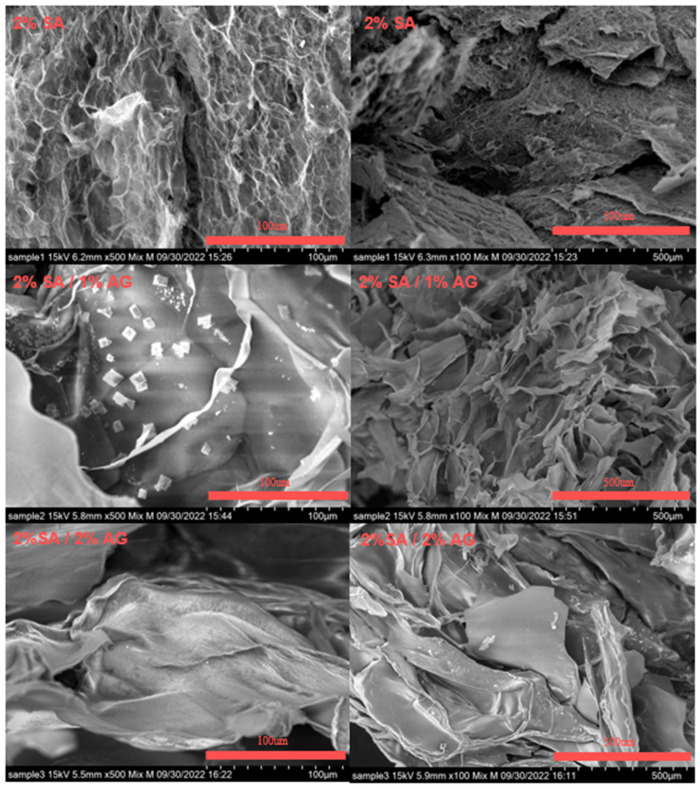
SEM images for different concentrations of inks. The compositions are listed in the figures.

The microstructure of TCC cryoprinted scaffolds using different inks was examined, specifically, 2% SA, 1% Agar/2% SA, and 2% Agar/2% SA. Figure 8 displays the scanning electron microscopy (SEM) images of the samples subjected to cryoprinting freezing treatment and subsequently stored at −80 °C. The results indicate a significant impact of agar addition on the microstructure in comparison to scaffolds printed solely with 2% SA. The SEM images illustrate differences in the internal structure and morphology of the scaffolds. Notably, the SEM images of the 1% Agar/2% SA and 2% Agar/2% SA samples exhibit larger pore sizes and higher porosity when compared to the 2% SA group. This observation emphasizes how the combination of agar, and the freezing process contributes to improved structural integrity of the AG/SA mixed gel scaffold.

The presence of agar has a significant influence on the microstructure of agar/alginate cryoprinted scaffolds, primarily by modifying the freezing and thawing behavior during the cryoprinting process. The incorporation of agar, along with the freezing treatment, results in increased porosity throughout the scaffold. During the freezing process, ice crystals are formed within the gel matrix. Upon thawing, these void spaces become filled with unbound water which contributes to the increased porosity. The higher concentration of agar further enhances this effect, leading to larger pore sizes and higher overall porosity in the agar-containing scaffolds. The increased porosity plays a crucial role in facilitating the diffusion of the crosslinker during the thawing process. The enhanced porosity allows the crosslinker to penetrate and distribute more effectively throughout the entire scaffold, resulting in a more uniform crosslinking. This improved diffusion and crosslinking contribute to the enhanced diffusivity of CaCl_2_ through the crosslinked structure.

Overall, these findings emphasize the beneficial effects of agar and the freezing process on the microstructure and porosity of the cryoprinted scaffold. The combination of agar and the freezing treatment leads to increased porosity, which in turn facilitates better crosslinking and improved structural integrity of the scaffold.

In summary, the casting experiments demonstrate that incorporating agar into the alginate ink brings several positive effects to the TCC technology. Firstly, agar undergoes gelation before freezing, effectively capturing, and preserving the desired printed structure before the alginate solidifies. This initial gelation step enhances the scaffold’s structural integrity. During the subsequent melting stage, the frozen agar loses its water-binding ability. This unique property of agar plays a crucial role in facilitating the crosslinking process. As agar loses its gel form and no longer retains water, the frozen agar domains enhance the diffusivity of the crosslinker. This increased diffusivity allows the crosslinker to permeate the structure more effectively as it melts, thereby promoting a successful crosslinking reaction. In essence, the addition of agar to the alginate ink not only helps preserve the printed structure before freezing but also assists in the efficient diffusion of the crosslinker during the subsequent melting phase. These combined effects contribute to the overall improvement of the TCC technology for advanced bioprinting applications.

### 2.3. Printing Test

**Figure 9:**
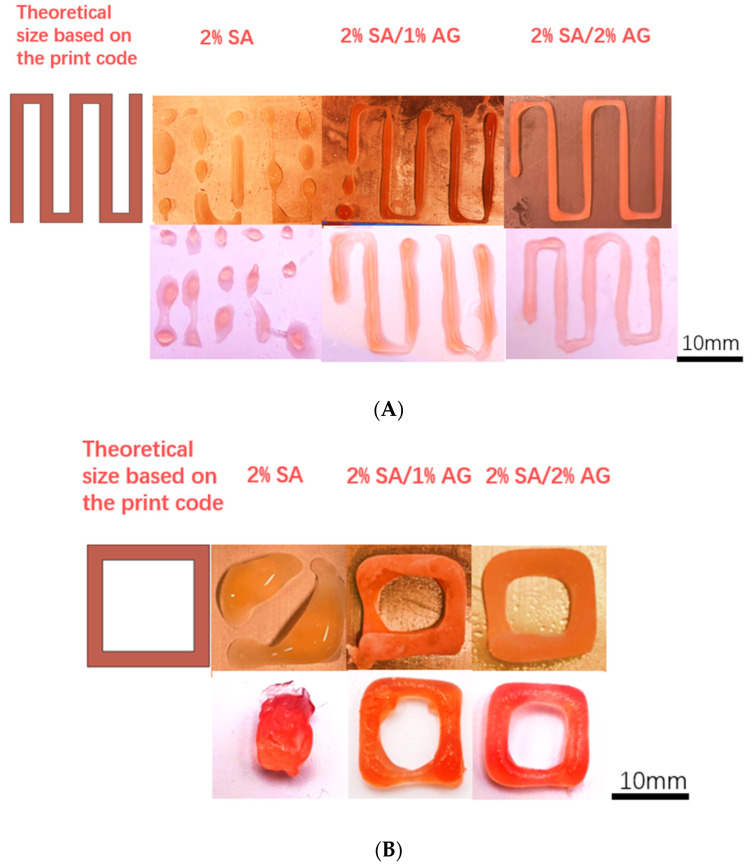

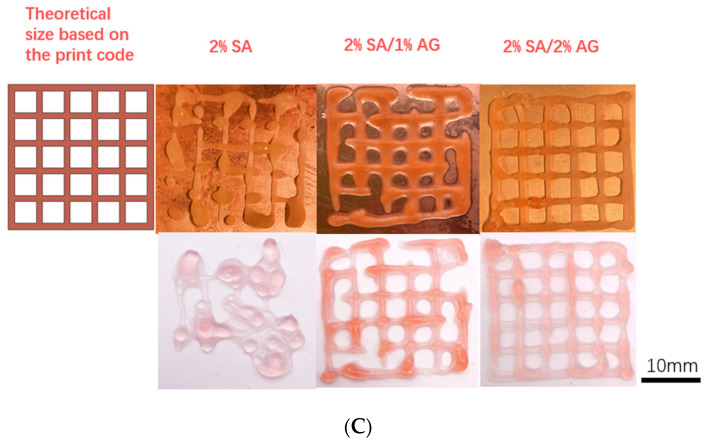
(**A**) The line structure print test. Top layer is the printing structure in frozen state; bottom layer is the printing structure after crosslinking. (**B**) The square print test. Top layer is the printing structure in frozen state; bottom layer is the printing structure after crosslinking. (**C**) The 5 × 5 grids print test. Top layer is the printing structure in frozen state; bottom layer is the printing structure after crosslinking.

To investigate the 3D printing properties of the TCC Alginate/Agar hydrogel ink, we utilized different concentrations of the SA/AG hydrogel inks to print various pre-designed shapes, including squares, straight lines, and 5 × 5 grids. All scaffolds were printed using a 16-gauge nozzle (Auerllcy, Seattle, WA, USA). The resulting printed structures can be seen in Figure 9. To enhance visibility, food dye was added to the ink solution at a ratio of 1 mL per 100 mL. The printing process took place on a −10 °C cold plate, and the temperature was carefully regulated using a cooling bath. After printing, the samples were transferred to a −80 °C freezer for 30 min and then subjected to crosslinking in a crosslinking bath of CaCl_2_ at room temperature.

**Figure 10:**
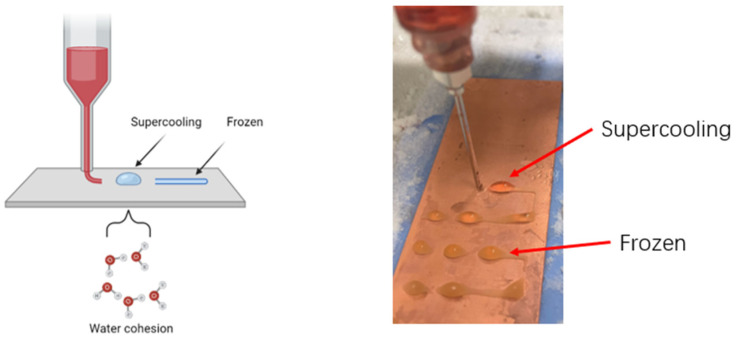
The supercooling phenomenon during cryoprinting.

Figure 9A shows that during the line printing test, the structures printed with 2% SA ink exhibited discontinuous lines with bead-like structures, while the structures printed with inks containing AG showed continuous lines. Among the three groups, the 2% SA/2% AG ink produced the best line quality. The occurrence of discontinuous bead-like structures can be attributed to several factors, with one primary factor being the supercooling state of the printing ink. Supercooling refers to the phenomenon where a substance remains in a metastable liquid state below its freezing temperature. In the case of sodium alginate ink, during extrusion from the nozzle, the printed structure remained in a supercooled liquid state with a temperature below its freezing point. The formation of bead-like structures can be attributed to the surface tension and cohesion of the ink, as shown in Figure 10. Supercooling is influenced by the sample volume and is a common occurrence in the early stages of cryoprinting with alginate. It is challenging to completely eliminate supercooling, as nucleation, which triggers freezing, is a probabilistic event that occurs at random after supercooling [34].

In contrast, the structures printed with ink containing agar did not exhibit visible bead-like structures. This can be attributed to the TCC cryoprinting process for SA/AG ink, which involves a phenomenon known as dual solidification (DS). In the DS process, the ink is extruded onto a cold plate. The first stage of solidification in DS is the gelation of agar, which occurs when the temperature drops below 30 °C. This initial gelation helps stabilize the printed structure before freezing occurs. Therefore, even if a supercooling state is present before freezing for SA/AG ink, it does not significantly affect the final structure as it does for SA ink. The presence of gelled agar in the ink provides stability and prevents the formation of bead-like structures. Another factor that contributes to the absence of bead-like structures is the viscosity of the ink. The SA/AG ink has a higher viscosity compared to the SA ink. The higher viscosity helps maintain the integrity of the printed lines and prevents the formation of discontinuous beads [28].

Overall, the combination of agar gelation, stabilization of the structure, and higher ink viscosity in the SA/AG ink contributes to improved line quality and the absence of bead-like structures in the printed shapes. During the printing test of square structures (Figure 9B,C), it was observed that the structure printed with 2% SA ink could not maintain its shape after printing. This can be attributed to the supercooling phenomenon and the lower viscosity of the SA ink, as discussed earlier. The supercooling state and lower viscosity contribute to the formation of irregularities and deformations in the printed structure. In contrast, the square structures printed with 2% SA/2% AG ink exhibited more uniform and well-defined edges compared to those printed with 2% SA/1% AG ink. The primary mechanism behind this improvement is the effect of agar on retaining the structure through cold gelation during the dual solidification process. The addition of agar to the SA ink, which leads to the dual solidification process of the TCC cryoprinting technique, contributes to the improved shape retention and structural integrity of the printed square structures, particularly in the case of the 2% SA/2% AG ink.

A common feature observed in all 3D printed structures is that the ink with 2% agar retains the structure better after melting and crosslinking. The mechanism behind this phenomenon is the same as that explained for the casting experiments. When the frozen alginate melts, the remnants of the previously frozen agar increase the diffusivity of the crosslinker. This enhanced diffusivity, combined with the melting process, assists in maintaining the desired shape of the printed sample.

The inclusion of 2% agar in the ink improves the retention of the printed structure after melting and crosslinking. The melted remains of the frozen alginate, along with the increased diffusivity of the crosslinker, work together to preserve the shape of the printed sample. As discussed in greater detail in the introduction, adding agar to alginate to enhance the rigidity and fidelity of alginate casts has been used in dentistry for making teeth prints since 1937 [25]. The combination of agar and alginate has also been utilized in the production of packaging films by casting [26]. The use of alginate/agar ink to increase ink viscosity and impart rigidity to 3D printed objects was reported in several papers [27,28,29]. However, to the best of our knowledge, this paper is the first report on the use of agar additives in conjunction with freezing.

The incorporation of agar into the alginate ink has proven to be advantageous for the TCC technology, yielding similar conclusions as the studies conducted on the casting process. The presence of agar in the ink offers several beneficial effects. Firstly, agar undergoes gelation before the freezing stage, effectively preserving the desired printed structure prior to alginate solidification. This initial gelation step contributes to the structural integrity of the printed scaffold. During the subsequent melting stage, the frozen agar loses its water-holding capability. This characteristic of agar plays a crucial role in promoting the crosslinking process. By no longer retaining water and transitioning from a gel to a liquid state, the frozen agar domains enhance the diffusivity of the crosslinker. This increased diffusivity allows for more efficient permeation of the crosslinker as the structure melts, facilitating a successful crosslinking reaction.

In summary, the addition of agar to the alginate ink within the TCC technology offers multiple advantages. It enables the retention of the desired printed structure through agar gelation before freezing and aids the crosslinking process during the melting stage by enhancing crosslinker diffusivity. These findings highlight the positive impact of agar in the TCC technology, paving the way for improved bioprinting applications.

### 2.4. Solid Scaffold Printing

Genuine biological tissues, such as the kidney, liver, heart, and pancreas, have a solid macrostructure [35]. In this study, a solid structure was 3D printed using an Agar/Alginate ink, through the TCC process. The printing process was conducted at a temperature of −10 °C. The following is the sequence for printing a bulk scaffold. For each layer, the perimeter was initially printed using a 16-gauge metal nozzle (Auerllcy, Seattle, WA, USA), while the interior was printed using an 8-gauge nozzle (Auerllcy, Seattle, WA, USA). The reason for using nozzles of different sizes in the printing process is to enhance the printing speed while maintaining resolution.

**Figure 11:**
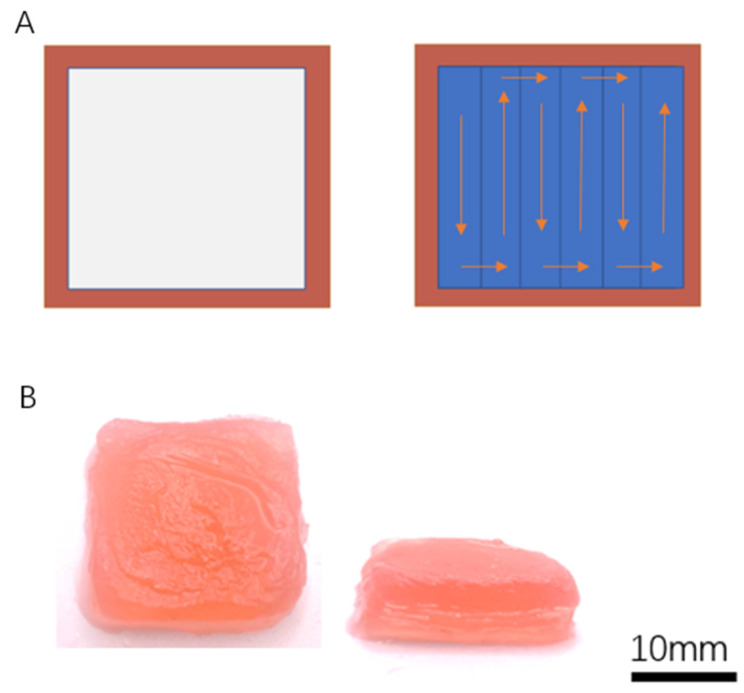
(**A**) The printing scaffold structure for a single layer. Left: the outer layer. Right: The printing strategy. (**B**) A cryoprinted solid scaffold.

Once the printing of the scaffold was completed, it was transferred to a −80 °C freezer for further freezing. After the freezing step, the scaffold was crosslinked in a room-temperature CaCl2 bath for 15 min, following the same procedure as the samples printed in the previous sections. Figure 11B displays a solid scaffold with dimensions of 2.5 cm × 2.5 cm × 0.6 cm, which was successfully printed using the TCC method.

### 2.5. Compression Test

**Figure 12:**
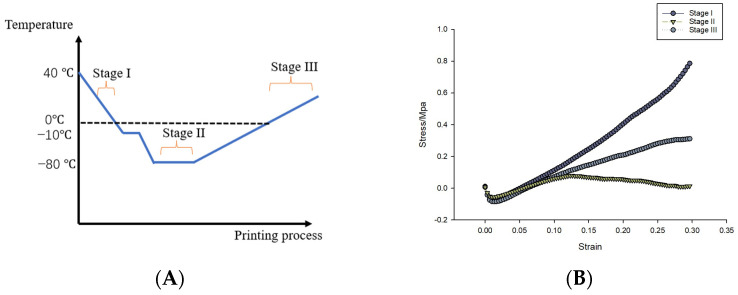
Compression test. (**A**) The temperature changing in the whole TCC process. (**B**) The compression test for the samples collected from different stages.

The compression test was conducted to evaluate the mechanical properties of the printing scaffold during different phases of TCC. Solid scaffolds, produced as described in the previous section, were used for the compression analysis. The results of this test are depicted in Figure 12. In this context, Stage I represents the state of the scaffold before freezing but after gelation. Stage II represents the scaffold’s condition after freezing but before the crosslinking process. Lastly, Stage III represents the scaffold following the crosslinking phase. To ensure reliability, all assessments were performed in triplicate. During the compression testing, samples from the different stages were maintained at room temperature.

The results of the compression test indicate a significant variation in the mechanical properties of the scaffold during the different stages of the TCC process. In Stage I, the stress–strain relation Young’s modulus is typical for that of an elastic agar gel. Stage II is for a frozen sample and illustrates the yield of the rigid frozen tissue. The Stage III figure also shows an elastic behavior; however, in this stage, the agar gel has suffered freeze ‘damaged’, and the Young’s modulus is that of crosslinked alginate. These results illustrate how the combination of agar and alginate affects the mechanical properties of the TCC product. It is particularly interesting to observe the transition between the Young’s modulus of elasticity of the agar gel to the Young’s modulus of elasticity of the crosslinked alginate through the freezing process. The function of the agar is critical, as it maintains the fidelity of the printed structure through the TCC process, as illustrated by the outcome of the casting and printing studies.

## 3. Conclusions

In conclusion, the utilization of SA/AG ink in temperature-controlled cryoprinting (TCC) has led to the successful fabrication of constructs with good shape fidelity, shape retaining ability, and high porosity. The SA/AG ink undergoes key phenomena during the TCC process that contribute to important outcomes. Firstly, the gelation of agar before freezing helps maintain the shape of the printed structure until freezing occurs. This ensures the preservation of the desired structure with high accuracy. During the melting stage, the agar loses its water-holding ability, thereby increasing the diffusivity of the crosslinker as the structure melts. This enhanced diffusivity facilitates a more effective and faster crosslinking process. The scaffolds produced using the SA/AG ink demonstrate remarkable structural stability during crosslinking and exposure to liquid, making them highly suitable for in vivo applications. Electron microscopy analysis confirms the presence of high porosity within the SA/AG-based TCC scaffolds, which explains the increased diffusivity observed after freezing agar-containing ink. The freezing process employed during cryoprinting plays a crucial role in enhancing the porosity of the scaffolds. In summary, the combination of Agar and Sodium Alginate in the ink formulation represents an effective approach for temperature-controlled cryoprinting. This formulation enables the fabrication of scaffolds with desirable properties, including superior shape fidelity, excellent structure retention, and high porosity. These findings open new possibilities for advanced tissue engineering and regenerative medicine applications.

Recently, our team demonstrated the feasibility of utilizing TCC technology to 3D cryoprinting viable Vero cells. This innovative approach employs an ink composed of a blend of alginate, collagen, and cells. Significantly, these cells demonstrate survival and growth after undergoing thawing and crosslinking processes, even within structures as complex as seven printed layers [12]. The primary objective of developing this technology was to manufacture 3D cell structures for investigating viral contamination, providing an alternative to prevalent 2D cell structure methodologies in this field.

Preliminary findings within our research group suggest that this technology offers versatility in layer count, effectively eliminating the previous dimensional constraints inherent in 3D printing of biological constructs. By combining the technology outlined in [12] with the discoveries elucidated in this paper, the prospect of bioprinting substantial organs while retaining structural precision and cellular vitality becomes attainable. A comprehensive review of 3D bioprinting research on the liver can be found in [36]. At present, our research efforts are focused on addressing the obstacles outlined in [36] concerning the 3D bioprinting of a liver. We aim to tackle these challenges by leveraging TCC technology, employing an ink formulation comprising alginate, gelatin, agar, and live cells. Our ultimate objective is to achieve the 3D bioprinting of an entire liver lobe, which will provide a foundational framework for investigating the effects of various pharmaceuticals on the human liver.

## 4. Materials and Methods

### 4.1. Agar/Alginate Solution

To prepare the gel solution, Agar (Sigma-Aldrich, St. Louis, MO, USA) and sodium alginate (Sigma-Aldrich, St. Louis, MO, USA) were dissolved in deionized (DI) water. The DI water was heated up to 100 °C using a heating plate (Corning Inc., Somerville, MA, USA) to dissolve the agar and sodium alginate powders. Once all the powder was dissolved, the solution was cooled to 40 °C. The study involved three compositions: 2% Sodium Alginate/2% Agar *w/w* (2% AG/2% SA), 2% Sodium Alginate/1% Agar *w/w* (1% SA/2% AG), and 2% Sodium Alginate *w/w* (2% SA).

### 4.2. Crosslinking Bath

A 2% calcium chloride (CaCl_2_) bath was prepared for crosslinking by dissolving 8 g of CaCl_2_ powder (Fisher Scientific, Hampton, NH, USA) in 400 mL of DI water. All frozen samples were stored in a −80 °C freezer prior to crosslinking. To initiate crosslinking, the samples were taken out from the freezer and fully submerged in the crosslinking bath at room temperature (23 °C) for 15 min. The crosslinking and thawing processes were continuously monitored, and significant events were recorded with corresponding timestamps.

### 4.3. Electron Microscope

Electron microscopy was performed on all the processed agar/alginate compositions. The sample being examined was initially rapidly frozen by immersing it in liquid nitrogen and then subjected to freeze drying using a 4.5 L −50 °C freeze dryer (Labconco FreeZone, Kansas City, MO, USA) for a duration of 2 days. The resulting freeze-dried sample was observed using a Hitachi TM-4000 scanning electron microscope (Hitachi, Chiyoda City, Tokyo, Japan) at various magnifications.

### 4.4. Experimental Systems

This paper presents the findings of multiple experimental studies focused on the TCC (temperature-controlled cryoprinting) process applied to agar/alginate solutions. Specifically, we investigate the deformation behavior, diffusivity, stability in a simulated human body environment, microscale structure, and printability of these solutions. Subsequently, we provide a detailed description of the devices employed in these experiments, both for the fabrication and testing of the samples. Two configurations were used in the study: (a) casting and (b) 3D printing.

### 4.5. Casts

A study with casting was designed to investigate the deformation behavior of samples composed of various agar and alginate compositions treated with freezing and immersion crosslinking. The samples were cast using the mold depicted in Figure 1, which features a top surface measuring 3.7 cm × 3.7 cm with rounded edges. To ensure consistent results, different agar/alginate inks with the same volume (3 mL) were injected into the mold container, ensuring an identical original cast for each sample. The ink solution was prepared at 100 °C and then cooled to 40 °C before casting. Once cast, all samples were cooled to room temperature to allow agar gelation and subsequently transferred to a −80 °C freezer for freezing. After freezing, the samples were collected from the mold container and immersed in a CaCl_2_ bath at room temperature for 15 min to initiate crosslinking. The process of thawing and crosslinking was closely monitored and recorded, aiming to gain insights into the underlying mechanisms.

### 4.6. Stability of Frozen/Crosslinked Cast Structures

To assess the structural integrity of tissue-engineered structures over extended exposure to a physiological environment, an experiment was conducted involving cast structures fabricated using different compositions of agar/alginate. The experimental setup mirrored the beforementioned post-crosslinking deformation study, utilizing the same casting geometry.

For each ink composition, two identical casts were prepared. One cast was stored in a sealed zip-lock bag at a temperature of 4 °C in a refrigerator, ensuring minimal evaporation. The second cast was immersed in a 1X phosphate-buffered saline (PBS) solution (Fisher Scientific, Hampton, NH, USA) at body temperature and placed in an incubator to simulate the conditions of the human body. All samples were left undisturbed for a duration of 24 h, after which their morphology was examined to evaluate any changes or degradation in their structure. This experiment aimed to determine the cast structures’ ability to retain their integrity in a liquid environment, simulating long-term exposure to the human body.

### 4.7. TCC Printing System

A temperature-controlled 3D cryoprinting (TCC) system was developed and constructed for this study. The printing component of the system was modified from an Ender 3 3D Printer, manufactured by Shenzhen Creality in Shenzhen, China. To enable cryoprinting, a freezing printing surface was designed and integrated into the printer. This surface was connected to a cooling bath supplied by Polyscience Inc., located in Phoenix, AZ. A cooling mixture comprising 50% ethanol and 50% water was employed in the bath, maintaining a temperature of −10 °C for the printing process. During printing, the syringe utilized for extrusion was equipped with a flexible heater from Omega, based in Norwalk, New Jersey. This heater was employed to sustain the syringe’s temperature at 40 °C, ensuring appropriate material flow and preventing unwanted gelation. Following the completion of the printing process, the frozen samples were carefully transferred to a −80 °C freezer manufactured by K2 Scientific, situated in Charlotte, NC, USA, for storage purposes.

### 4.8. Compression Test

Compression tests were carried out using a TA. XT plus 100 texture analyzer (Stable Microsystems Ltd., TA-XT2i, Godalming, UK). The compression tests utilized a 5 mm diameter cylindrical probe. During the tests, the samples were compressed by 2 mm at a speed of 0.2 mm/s for a duration of 10 s. To ensure accuracy and reliability, three repetitions of the compression test were performed for each group configuration or sample condition. This approach allowed for consistent and reproducible measurements during the compression analysis.

### 4.9. Statistical Analysis

In the shape retainability test and compression test analyses, statistical analysis was conducted using a one-way analysis of variance (ANOVA). This test allowed for the comparison of means across multiple groups or conditions. To determine significant differences between the groups, a post hoc analysis known as Duncan’s multiple range test (DMRT) was employed. The DMRT helps identify specific pairs of groups that exhibit significant differences while maintaining a 95% confidence level. The statistical analysis, including ANOVA and DMRT, was performed using SigmaPlot 15.0 software.

## Data Availability

Data will be made available on request.
